# Cross-country Association of Press Freedom and LGBT freedom with prevalence of persons living with HIV: implication for global strategy against HIV/AIDS

**DOI:** 10.1186/s41256-018-0061-3

**Published:** 2018-02-09

**Authors:** Xinguang Chen, Amy L. Elliott, Shuang Wang

**Affiliations:** College of Public Health and Health Professions and College of Medicine, University of Florida, Gainesville, Florida USA

**Keywords:** HIV/AIDS epidemiology, Press freedom, LGBT freedom, Core values, Global health

## Abstract

**Background:**

Human behaviors are affected by attitudes and beliefs, which in turn are shaped by higher-level values to which we have ascribed. In this study, we explore the relationship between two higher-level values, press freedom and LGBT freedom, and HIV infection with national data at the population level.

**Methods:**

Data were the number of persons living with HIV (PLWH, *n* = 35,468,911) for 148 countries during 2011-15, press freedom index (PFI) determined by the Reporters Without Borders, and LGBT freedom index (LGBT-FI) based on laws regulating same-sex relationships and expression. PLWH prevalence (1/1000), PFI and LGBT-FI were mapped first. Multiple regression was thus used to associate the logarithm of PLWH prevalence with PFI, LGBT-FI and PFI × LGBT-FI interaction, controlling for per capita GDP and weighted by population size.

**Results:**

Global prevalence of PLWH during 2011-15 was 0.51 per 1000 population. The prevalence showed a geographic pattern moving from high at the south and west ends of the world map to low at the north and east. Both PFI and LGBT-FI were positively associated with PLWH prevalence with a negative interaction between the two.

**Conclusions:**

More people are infected with HIV in countries with higher press freedom and higher LGBT freedom. Furthermore, press freedom can attenuate the positive association between levels of LGBT freedom and risk of HIV infection. This study demonstrated the urgency for and provided data supporting further research to investigate potential cultural and socioecological mechanisms underpinning the complex relationship among press freedom, LGBT freedom and HIV infection, with data collected at the individual level.

**Electronic supplementary material:**

The online version of this article (10.1186/s41256-018-0061-3) contains supplementary material, which is available to authorized users.

## Background

The United Nations, through its joint program on HIV/AIDS (UNAIDS), set an ambitious goal to end the AIDS epidemic by 2030 [1]. To achieve this goal, global efforts have been devoted to investigating factors related to the HIV epidemic in order to adapt the most effective strategies to identify and treat those individuals infected with the AIDS virus [2–4], prevent the spread of the virus [5–9], and/or to develop a cure for AIDS [10, 11]. Despite these rigorous efforts and great progress made in combatting the disease, worldwide there are still an estimated 37 million persons living with HIV (PLWH), with approximately 20% living in South African countries [12, 13].

Across all ages and racial groups, media is often the first and sometimes the only information source regarding HIV/AIDS [14, 15]. HIV-related media has been used as an effective tool to spread HIV-related knowledge and prevention skills, and to promote HIV prevention and treatment-seeking behaviors [16]. However, media can also have a negative effect on people’s behaviors, worsening the HIV epidemic. For example, taking advantage of the freedom of expression, Thabo Mbeki (the former president of South Africa) ran a campaign that discouraged use of antiretroviral drugs, saying that they were too expensive and might be toxic and that traditional medicines were better [17]. Hundreds of thousands of people are estimated to have lost their lives due to this campaign [17].

Human behaviors, attitudes, and beliefs may present a challenge to ending the global AIDS epidemic. Despite increased knowledge regarding HIV infection and safer sex practices from social marketing and other intervention measures, a sizable number of persons still make decisions that put themselves at risk for HIV infection. For example, given the awareness of the protective effect of condom in HIV prevention [18] and the knowledge of high personal risk of HIV infection [19–21], a large number of LGBT (lesbian, gay, bisexual and transgender) individuals, particularly MSM (men who have sex with men), still choose not to use protective measures to avoid HIV infection during sexual encounters [22–25]. Similarly, studies on HIV prevention interventions commonly report that many individuals at high risk for HIV infection do not think condoms necessary, and are reluctant to use a condom during sex [22, 26, 27].

When a person appreciates his/her own freedom of expression, he/she may be totally unaware that his/her behaviors are in fact determined, to a substantial extent, by his/her attitudes and beliefs [28–31], while the attitudes and beliefs are, in turn, shaped by higher-level values and norms to which he/she has ascribed [31–33]. Media is one of the primary sources that shape our behaviors by spreading and promoting specific social norms, cultural values, and personal beliefs, attitudes and behaviors such as freedom of expression, freedom of action, and LGBT freedom. Therefore, a relationship between HIV infection and free expression, particularly LGBT freedom, may provide evidence at the macro level supporting the role of media in HIV transmission [31–33]. Findings from such studies will also provide data needed to enhance the current global strategy for HIV/AIDS control by appropriately emphasizing the role of media. To date, no such study has been reported in the literature.

The understanding of freedom in general and LGBT freedom in particular differ for persons who live in countries where these values were adapted recently (e.g., Russia and the three newly independent Baltic countries from the former Soviet Union during 1989-91, and China since it adapted the Open Policy in 1978) than those in many western countries where the values have been a primary part of the mainstream culture (e.g., most West Europe and North American countries). Compared to countries with a long history of appreciation of the value of freedom, countries that have recently adapted press freedom may have a limited interpretation of freedom. The spread of freedom in the media in these countries could be small in scope and the understanding of freedom could be fragmented and biased.

The differences in understanding the value of free expression, including LGBT freedom, may result in different sexual practice, altering the risk of HIV infection. For example, people in the countries where the value of freedom has been introduced recently may be more passionate about the value of freedom. Many LGBT individuals in these countries may simply consider sexual freedom in an “absolute” sense. These individuals could be more likely to think it is their own right to form sexual relationships and to engage in sex with any person(s) of their preference, treat condom use during sex as a personal choice, and ignore the responsibility of not passing the HIV virus to their partners.

On the contrary, people who live in countries with a long history of press freedom may have a more systematic understanding of LGBT freedom via long-term exposure to and/or participation in open and systematic discussion over the matter. While pursuing personal freedom, LGBT individuals in these countries may be more likely than those in other countries to take prevention measures, considering both the modern value of personal sexual freedom and the traditional values of others, as well as families and the society as a whole [34]. If this hypothesis is true, the relationship between LGBT freedom and HIV infection will be stronger in most non-western countries, where the value of freedom was adapted recently; while the same relationship will be weaker in most western countries which claimed to be founded on the value of freedom. However, no published study in the literature has examined the potentially interactive effect of freedom of expression with LGBT freedom on HIV infection.

This study attempted to assess if the prevalence of HIV infection (UNAIDS data) is associated with levels of press freedom (data from Reporters Without Borders) and LGBT freedom (laws related to same-sex relationships and expression) with country-level data. We hypothesize a positive relationship at the country level between HIV infection and press/LGBT freedom, and furthermore the relationship between LGBT freedom and HIV infection is attenuated by press freedom.

## Methods

### Data sources and variables

#### Data on number of persons living with HIV by country

We elected to use PLWH data available in the most recent years by country. Data were obtained for a total of 148 countries. Of these countries, 2015 data were acquired for 111 (75%) from the UNAIDS Data Hub [13, 35] and governmental reports (UK, China and Laos) [13, 35, 36]. For the remaining 37 countries, data available from the most recent years (from 2011 to 2014) were derived from other sources. Of these countries, data for 27 countries were acquired from the HIV/AIDS Country Profile by the WHO Regional Office for Europe [37], data for 7 countries from the Country Progress Report of UNAIDS [38], data for Estonia from WHO’s Evaluation Report [39], Guinea-Bissau from the UNICEF (United Nations International Children’s Emergency Fund) report [40], and U.S. data from CDC’s (Center for Disease Control and Prevention) [41]. Potential under-detection of HIV infections in the low- and middle-income countries in the reported data were adjusted by UNAIDS and WHO.

#### Data on population by country

Total population for the 148 countries (per 1000 persons) was acquired from the database hosted by the World Bank [42]. These data were processed by the World Bank through the United Nations Population Division. The population data for each country was matched with PLWH data by year and was used to compute country-specific prevalence of PLWH for global mapping and for statistical analysis.

#### Data on press freedom index (PFI)

PFI was the predictor variable. Data for PFI by country for the 148 countries in 2015 were included. PFI data were derived from the annual report by Reporters Without Borders [43]. The index was established with original data from a cross-country survey conducted by the Reporters Without Borders. The survey consists of 87 questions, assessing six aspects of press freedom, including (a) pluralism, (b) media independence, (c) environment and self-censorship of journalism, (d) legislative framework related to free press, (e) press transparency, and (f) infrastructure and abuses against journalists. Typical questions include: “In your country, do any of the following exist? (1) Privately owned print press, (2) privately owned television, (3) privately owned radio stations.” “Does the government monitor or threaten journalists?” “Can citizens directly and freely contact journalists, with no government controls or monitoring, especially in order to provide information?”

The public available PFI data were created by the Reporters Without Borders using a complex weighted method of the six domains defined for press freedom using data from the 87 survey questions. The theoretical range of the original PFI varied from 0 to 100 with higher scores indicating diminished freedom of the press. The recorded scores for 2015 ranged from 7.52 to 84.86. For the convenience of result interpretation, in this study we reversely coded the original PFI by subtracting the reported index of each country from the maximum value of 84.86. The recoded scores ranged from 0.00 to 77.34 with larger values indicating greater press freedom. For efficient regression analysis, we rescaled the PFI into the range of 0.00 to 7.73 by dividing the recoded scores with10.

#### Data on LGBT freedom index (LGBT-FI)

This index was created to measure LGBT freedom based on laws regulating same-sex marriage and expression in different countries across the globe. The LGBT-FI was created using data from Wikipedia Commons that categorizes countries worldwide into 10 groups based on their laws related to protection of LGBT relationships and expression [44]. For better presentation and statistical analysis (not too few countries in one group), a five-level LGBT-FI was created with 1 = countries with death penalty for same-sex intercourse (such as Iran and Yemen), 2 = countries with imprisonment (such as Egypt and Lebanon), even up to life in prison (such as India and Pakistan) for same-sex intercourse, 3 = countries where expression and practice of same-sex relationships are not recognized (e.g., most East European countries), restricted (e.g., Russia) or illegal (e.g., Algeria), 4 = countries where same-sex relationships including civil union (e.g., China) and cohabitation (Pacific Island countries) are recognized or legally allowed; 5 = countries where same-sex marriage is recognized (e.g., part of Mexico) or protected by laws (e.g., the United States and Western European countries). The arbitrary index scores provide a semi-quantitative ranking measure for statistical analysis.

### Covariates

As countries that value press freedom are predominantly highly industrialized with high incomes, the 2015 per capita GDPs of all the countries were included. This variable was used as a covariate in statistical analyses to assess the complex relationships among PFI, LGBT-FI and HIV prevalence. Other covariates, such as religion and other cultural factors were not included due to several reasons. First in this study, individual countries were the unit of analysis; but few countries, if any, possess only one religion or a single-component culture. It is a technical challenge to quantify such factors as covariates for analysis. Second, press freedom and LGBT freedom were used as the distal and overarching variables in predicting the outcome HIV prevalence. Variables like religion between these distal variables and HIV infection are epidemiologically not confounders; therefore, they were not considered as covariates.

### Data processing and statistical analysis

A database was established by manually entering all the derived information, including the number of PLWH, population size, PFI, LGBT-FI, and per capita GDP by country. Data used for this analysis are included in the Additional file 1. Data quality was examined carefully against the official records before they were analyzed. The prevalence of PLWH per 1000 population was computed as the ratio of the total number of PLWH over the total population and was used as the outcome variable.

To provide a visual observation, the estimated PLWH prevalence the reverse-coded PFI, and the derived LGBT-FI were plotted by quintile on the most updated world map using the software ArcGIS, version 10.3.1. In drawing the maps for HIV prevalence and PFI, the standard quantile protocol from ArcGIS was used to create a five-level scale such that the number of countries in each group was about the same for efficient visual presentation. In mapping LGBT-FI, the coded freedom index numbers 1-5 were used directly without re-categorization.

Multiple linear regression analysis was conducted to associate PFI, LGBT-FI and their interaction with PLWH prevalence by country. Since the distribution of PLWH prevalence by country was not normal, a logarithm transformation was performed. With this approach, the analytical results regarding the relationship between PFI/LGBT-FI and HIV infection could be interpreted meaningfully by taking an anti-logarithm calculation of the estimated regression coefficient. In addition to F-tests and *R*^*2*^ for assessing the data-model fit, residual analysis was also conducted. A normal distribution of the residuals around zero without significant dispersion was used as an indication of good data-model fit. To ensure robust regression results, outliers were tested using Cook’s D, with the criterion of D < 4 indicating no outliers.

Regression analysis was conducted for PFI and LGBT-FI separately first, then together, and lastly with the term PFI × LGBT-FI interaction included. In each of these analyses, we started a simple model with no covariate, followed by a more complex model with per capita GDP (in $10,000) added as a covariate. GDP was added to regression models because most countries with higher PFI and LGBT-FI were western and developed, which may cofound the relationship between the predictors and the outcome variable. In addition, since countries in this study were used as the unit of analysis, population size of individual countries was used as weights in all regression models. The analyses were conducted using the commercial software package SAS, version 9.4 (SAS Institute, Cary, NC).

## Results

### General information

Among the 148 countries included in this analysis, 35,468,911 PLWH were reported. The total population of these countries represents 96.7% of the world population. The estimated overall PLWH prevalence per 1000 population [95% CI] for the 148 countries was 0.511 [0.429, 0.591]. The estimated PLWH prevalence by countries varied from the lowest of 0.06 for Bangladesh to the highest of 170.94 for Swaziland, with the median of 2.68 for Indonesia.

### Geographic distraction of PLWH across the globe

**Fig. 1:**
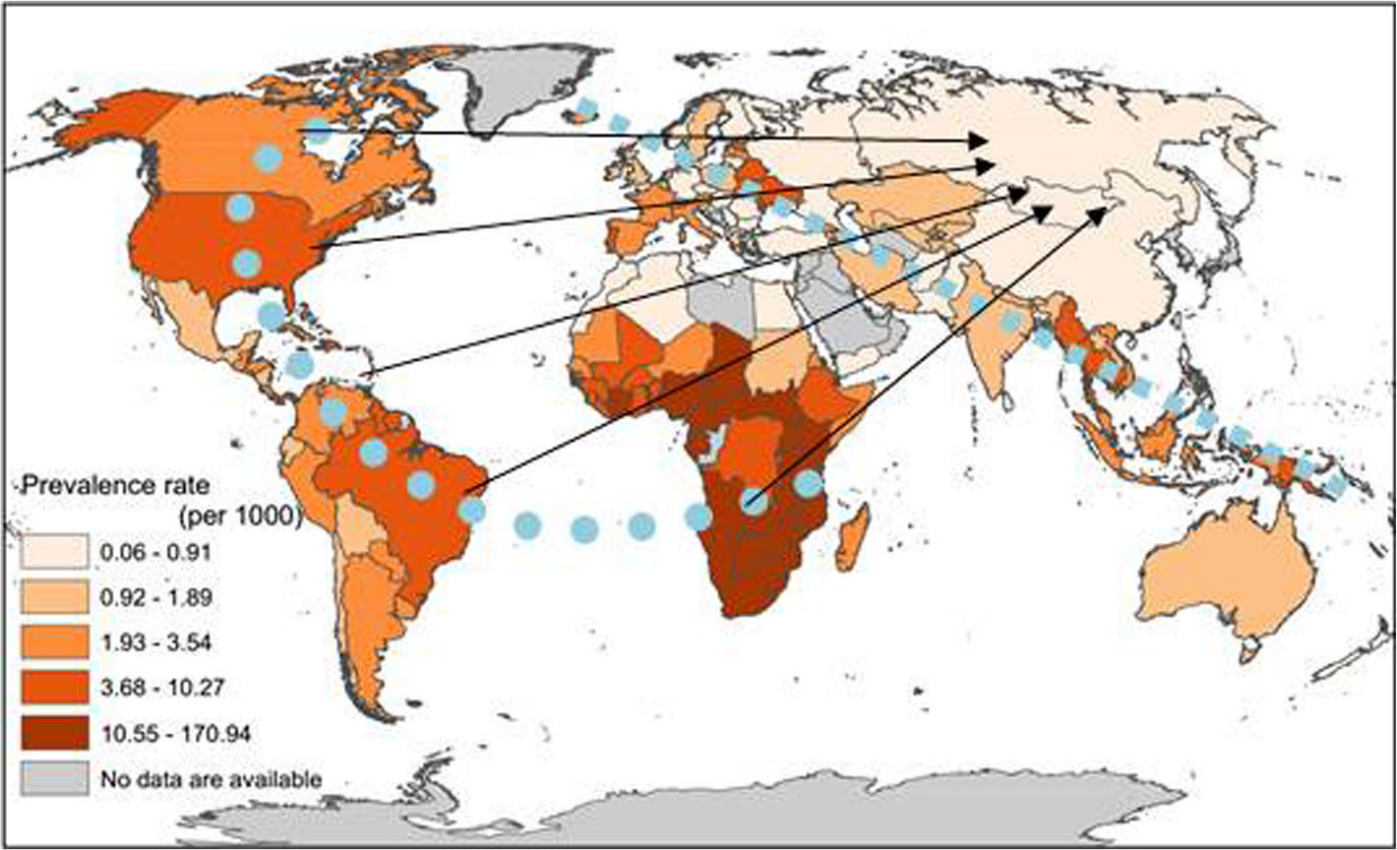
Global patterns of the prevalence rates (1/1000 population) of persons living with HIV (PLWH), 2011-2015 (Source: Created with data from UNAIDS and other sources)

Figure 1 depicts the global pattern of PLWH by country in or around 2015. PLWH prevalence per 1000 population by country from high to low formed a trend from the southwest edge of the map to the northeast edge of the map (as the arrows indicate).

A closer examination of the map revealed two additional patterns. (1) A C-shaped band of high PLWH prevalence (dotted light blue line). It started from Canada and the United States at the top left of the map, stretching downward to South American countries, moving right or eastward to cross the Atlantic and ending in Central and Southern African countries. (2) A narrow and linear band of high PLWH prevalence (dashed light blue line). It started from Iceland, passing through most European countries (including countries that came from the former Soviet Union, such as Estonia, Latvia, and Ukraine), gently touching Iran and India, and ending in Southeast Asia (particularly Burma, Thailand, and Papua New Guinea).

Globally, countries with low PLWH prevalence were mostly in (1) the Middle East and North Africa (MENA), including Morocco, Algeria, Egypt, Sudan, and Yemen; and (2) in the east, including China, Mongolia and Russia.

### Geographic patterns of the press freedom index

**Fig. 2:**
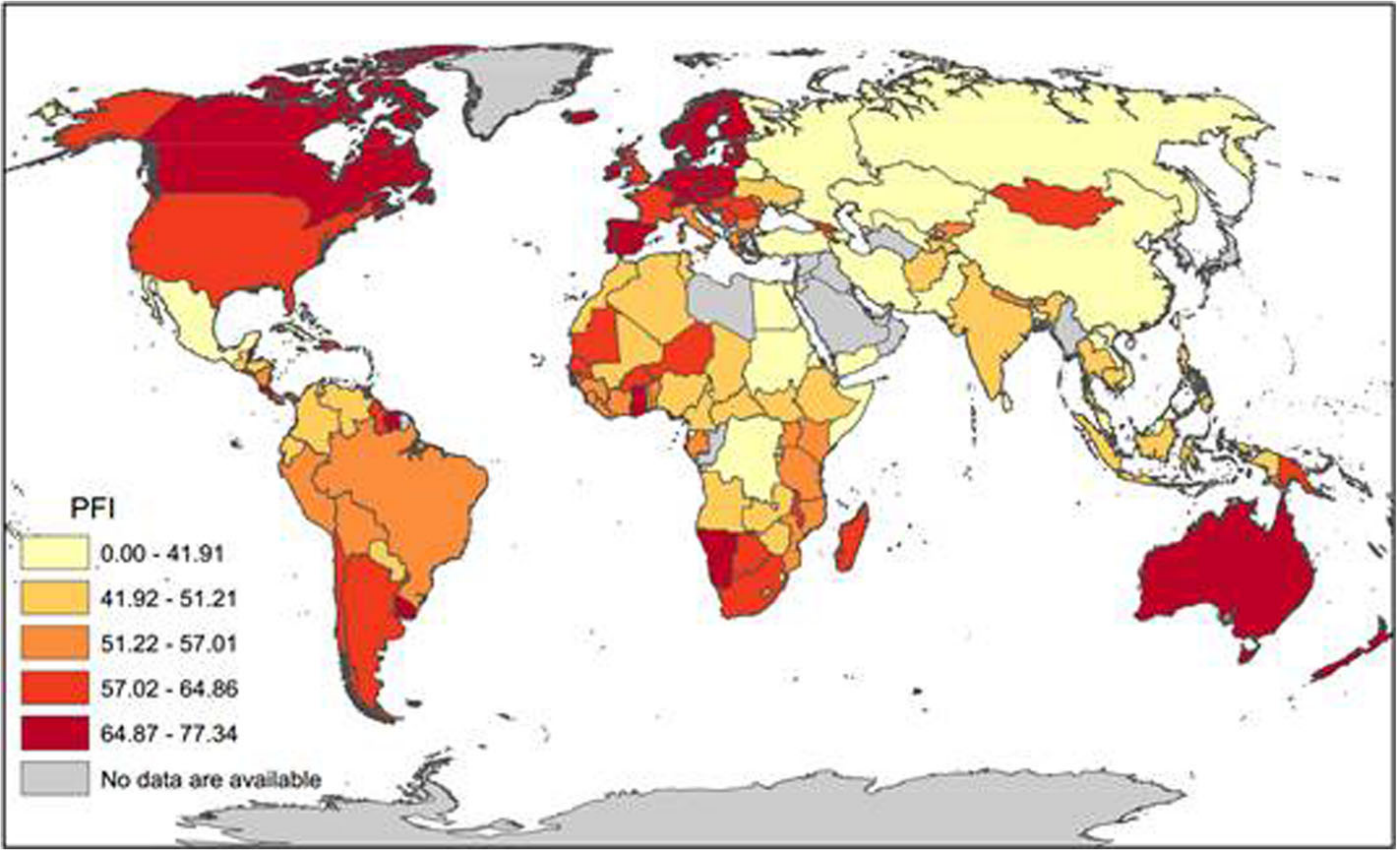
2015 Global pattern of Press Freedom Index with larger numbers indicating higher levels of press freedom (Source: Created using data from the Reporters Without Borders)

Likewise, Fig. 2 maps the PFI in 2015 across the globe. The mean PFI was 32.6 (SD = 16.2) for the 148 countries. In the map, countries marked with light yellow were the lowest and dark red the highest with regard to level of press freedom. PFI by country formed an overall pattern of high for America on the left to a mixture in the middle for Europe and Africa and low on the right for Asia. Likewise, a closer examination of the map revealed an extended C-shaped curve of high PFI starting in European countries, stretching back to North American, passing through South America and South Africa, and ending in Australia and New Zealand.

The mega patterns of PFI in this figure show certain similarity to those for PLWH in Fig. 1, suggesting a positive relationship between the two. For example, by comparing Fig. 1 with Fig. 2, one can see that countries with higher PFI also had higher PLWH prevalence, such as Estonia, Iceland, America, Dominic Republic, Namibia, South Africa, and Madagascar. Similarly, several typical countries characterized with both low PFI and low PLWH prevalence were China, Russia, Pakistan, Turkey, Egypt, Sudan and Congo.

### Geographic patterns of the LGBT freedom

**Fig. 3:**
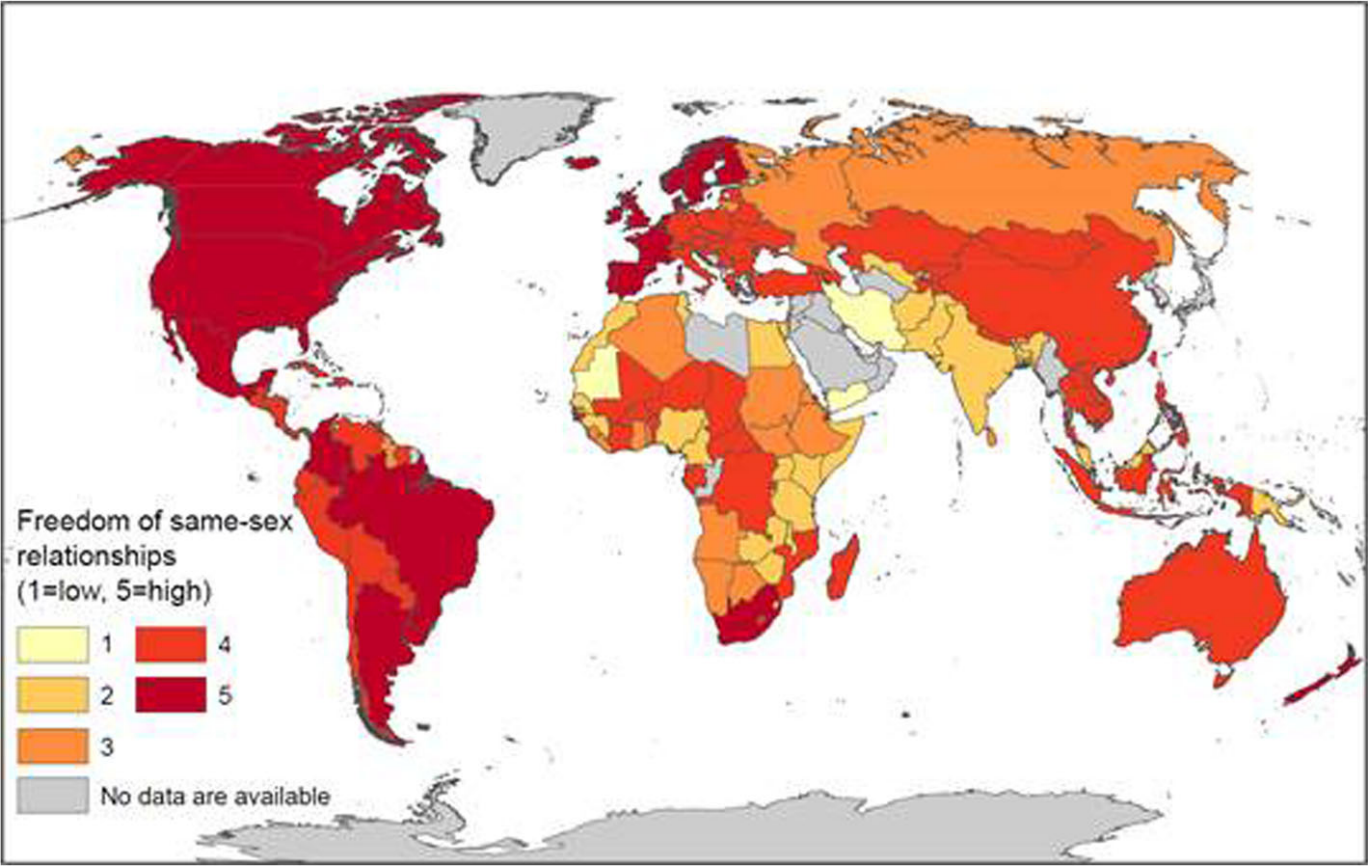
Global pattern of LGBT Freedom with larger numbers indicating higher levels of LGBT freedom (Source: Created with data from Wikipedia Commons on laws related to same-sex relationships and expression by country. See text in 2.1 for a detailed definition)

Figure 3 presents the global pattern of LGBT-FI. Among the total 148 countries, 5 (3.4%) had an LGBT-FI = 1, such as Iran and Yemen; 23 (15.5%) had an LGBT-FI = 2, such as Egypt and Lebanon; 25 (16.9%) had an LGBT-FI = 3, such as many Eastern European countries, Algeria and Sudan; 74 (50%) had an LGBT-FI = 4, such as Chile, China, and many Pacific Island countries; and 21 (14.1%) had an LGBT-FI = 5, such as the United States, Canada, Brazil and South Africa.

### Results from population-weighted regression analysis

Results from multiple regression analysis indicated that our data satisfactorily fit the proposed models. Regression diagnostic tests indicated no outliers for all regression models using the criteria of Cook’s D < 4. The distribution of residuals of these models was not significantly different from normal and was centered at zero. F-tests indicated that all models were statistically significant at either the *p* < 0.05 or *p* < 0.01 level.

Results in the top panel of Table 1 indicate that PFI was significantly and positively associated with HIV prevalence by country after controlling for GDP. The regression coefficient b [95% CI] was 0.3707 [0.2475, 0.4939], equivalent to an increase [95% CI] in HIV prevalence per 1000 population by 1.45 [1.28, 1.64] for every 10-point increase in PFI. Results in the second panel of Table 1 indicate no significant association at the *p* < 0.05 level between LGBT-FI and HIV prevalence with and without controlling for GDP. When both PFI and LGBT-FI were analyzed together, results in the third panel of the table indicate that PFI was highly statistically significant (*p* < 0.01) with b [95% CI] = 0.4024 [0.2746, 0.5303], equivalent to an increase of 1.50 [1.28, 1.70] in PLWH prevalence per 1000 population for every 10-point increase in PFI. In addition, LGBT-FI became marginally (*p* = 0.0919) significant with b [95% CI] = 0.1926 [− 0.0318, 0.4170], equivalent to an increase of 1.21 [0.97, 1.51] in PLWH prevalence per 1000 population with one level of LGBT-FI increase.

**Table 1:** Results from multiple regression using the Press Freedom Index to predict the prevalence rates of persons living with HIV in countries across the globe

Models	Parameter Estimate	Standard Error	95% CI	*P* value
Association of PFI				
Model I (*R*^*2*^ = 0.1887)				
Intercept	−0.88341	0.2469	−1.3222, − 0.33460	< 0.01
PFI (0-78)	0.3239	0.0561	0.2130, 0.4349	< 0.01
Model II (*R*^*2*^ = 0.2068)				
Intercept	−0.9017	0.2553	−1.4066, − 0.3968	< 0.01
PFI (0-87)	0.3707	0.0623	0.2475, 0.4939	< 0.01
Per capita GDP ($10 k)	−0.0137	0.0077	−0.0290, 0.0012	> 0.05
Association of LGBT-FI				
Model I (*R*^*2*^ = 0.006)				
Intercept	0.3954	0.3304	−0.2576, 1.0485	> 0.05
LGBT-F	0.0232	0.0952	−0.1649, 0.2113	> 0.05
Model II (*R*^*2*^ = 0.012)				
Intercept	0.4921	0.3726	−0.2445, 1.2288	> 0.05
LGBT-F	−0.0195	0.1212	−0.2590, 0.2200	> 0.05
Per capita GDP ($10 k)	0.0463	0.0978	−0.1415, 0.2377	< 0.05
Association of PFI and LGBT-FI				
(*R*^*2*^ = 0.223)				
Intercept	−1.5679	0.4673	−2.4920, −0.6438	< 0.01
PFI	0.4024	0.0646	0.2746, 0.5303	< 0.01
LGBT-F	0.1926	0.1135	−0.0318, 0.4170	> 0.05
Per capita GDP ($10 k)	−0.2396	0.0098	−0.0433,-0.0046	< 0.05
Interaction of PFI and LGBT-FI				
(*R*^*2*^ = 0.270)				
Intercept	−7.1081	1.7650	−10.5987, −3.6174	< 0.01
PFI	1.6359	0.3850	0.8745, 2.3972	< 0.01
LGBT-F	1.6007	0.4473	0.7161, 2.4854	< 0.01
PFI * LGBT-F	−0.3136	0.0966	− 0.5046, − 0.1226	< 0.01
Per capita GDP ($10 k)	− 0.0143	0.0099	− 0.0339, 0.0053	> 0.05

Note: Prevalence rates of persons living with HIV by country were transformed by taking a natural logarithm. Population size of individual countries was included as regression weights

**Fig. 4:**
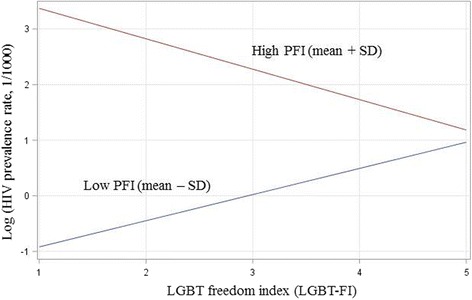
Interaction between press freedom index (PFI) and LGBT freedom index (LGBT-FI) in association with PLWH prevalence across 148 countries in the world (Source: Created with data from the UNAIDS, Doctors Without Borders, and Wikipedia Commons)

Regression results in the bottom panel of Table 1 indicate significant effect of PFI with the regression coefficient b [95% CI] = 1.6359 [0.8745, 2.3972] (*p* < 0.01) and LGBT-FI with b [95% CI] = 1.6007 [0.7161, 2.4854] (*p* < 0.01), equivalent, respectively, to an increase of 5.13 [2.40, 10.99] in PLWH prevalence for every 10-point increase in PFI and an increase of 4.96 [2.05, 12.01] in PLWH prevalence for each level of LGBT-FI increase. In addition, there was a significant and negative interaction (*p* < 0.01) between PFI and LGBT-FI with the regression coefficient b [95% CI] = − 0.3136 [− 0.5046, − 0.1226]. This effect was equivalent to a reduction of 0.97 [0.95, 0.99] in PLWH prevalence with PFI increasing every 10 points and LGBT-FI moving up one level. Figure 4 further depicts the interaction between PFI and LGBT-FI in affecting the prevalence of PLWH. Results in the figure show that LGBT-FI was negatively associated with PLWH prevalence in countries with higher PFI (like many European and North American countries), but positively associated with PLWH prevalence in countries with lower PFI (like China and Russia).

## Discussion and conclusions

Motivated by the goal to end the AIDS epidemic by 2030, this study examined the global associations between PLWH prevalence and two measures of the freedom of expression for 148 countries in the world. One freedom measure is the Press Freedom Index compiled and published by the Reporters Without Borders; and another freedom measure is the LGBT freedom index created based on laws regulating same-sex relationships and practice in different countries. Data on PLWH were based on the statistics from UNAIDS and governmental agencies in charge of HIV prevention and control. In addition to revealing the geographic mega trends in the HIV epidemic through global mapping, multiple regression analyses were conducted to quantify the association between PLWH prevalence and the two freedom-expression measures, weighted by population size and controlled for GDP as a potential confounder.

Great progress has been made in a number of HIV/AIDS-related research areas including biological, psycho-behavioral and socioeconomic factors. However, research on HIV risk and preventive behaviors and cultural values and social norms are limited [31, 45–47], furthermore, few studies, if any, have investigated the role of core cultural values such as press freedom and LGBT freedom as they are related to the HIV epidemic. This exploratory study provided data at the global level, the first time, filling in this knowledge gap. Findings of this study also demonstrate the urgency for and provide important preliminary evidence supporting more in-depth multi-country studies with data collected from individual participants.

During the process when we conducted this study, we realized the complexity to characterize the relationship between freedom of expression and HIV infection across countries in the world. When analyzed separately, levels of press freedom were significantly associated with prevalence of HIV infection and LGBT freedom was not. However, when the two variables were analyzed together in one model, both were positively associated with HIV infection at the country-level.

Furthermore, when a more realistic model with the interaction between press freedom and LGBT freedom being added, there were increases in the strength of the association of the PLWH prevalence with both press freedom and LGBT freedom with further reductions in type I error (smaller *p* values). The results suggest an increase of approximately 5.13 more HIV infections per 1000 population in a country along with every ten-point increase in press freedom index and an increase of 4.96 HIV infections with LGBT freedom index moving up one level, respectively. Above the two independent associations, the negative interaction between press freedom and LGBT freedom suggests a reduction of 0.97 HIV infections per 1000 population along with one level up in the LGBT freedom in countries with high press freedom; but a increases of 0.97 HIV infections along with one level up in LGBT freedom in countries with low press freedom. These results provide evidence for clarifying the contradictory findings from other studies on whether press freedom is a risk factor for HIV infection [48–50].

Findings of our study bear several important implications. First, we want to point out the negative interactions between press freedom and LGBT freedom. This finding indicates that press freedom can alter the risk effect of LGBT freedom on HIV infection at the country level. In countries with lower press freedom, LGBT freedom is associated with increases risk of HIV infection; while in countries with higher press freedom, LGBT freedom is associated with reduced risk of HIV infection. Results from our analysis suggest that moving up one level of LGBT freedom will result in a reduction of 1.85/1000 in the number of PLWH per 1000 population in high press-freedom countries with PFI greater than the mean plus one standard deviation (SD), while a one level increase in LGBT freedom will be related to 2.25/1000 increase in PLWH per 1000 population in low press-freedom countries with PFI less than the mean minus a SD. Although the effect is substantial, the exact mechanisms underlying the interaction between press freedom and LGBT freedom are unknown. Based on the evidence that countries with high PFI are mostly those with a long history of emphasis on press freedom, we may assume that people who pursue LGBT relationships and practice in these countries may have received better education on the importance of both safer sex practices and personal freedom and choices than those in the countries where press freedom and freedom of expression have been adapted more recently. In-depth multi-country studies with data at the individual level are needed to test this hypothesis.

Second, we cannot ignore the impact of both press freedom and LGBT freedom on the risk of HIV infection. The positive impact of press freedom on HIV infection has been reported in the literature [48]. Findings of our study provide data from a different source supporting the conclusion that MSM consist of a subpopulation with the highest risk for HIV infection [19–21]. In countries that newly adapted the value of freedom such as China, along with the introduction of greater freedom for sexual minorities, including LGBT, a rapid increase in new HIV infections, primarily among young MSM has been observed [21, 51]. Therefore, a potential effort to strengthen the current strategy for HIV prevention and control in these countries would be to allow for broader discussion about what free expression and sexual freedom really mean. When a person pursues sexual freedom, he/she must consider the consequences of and be responsible for his/her own behavior, and care about his/her partners, family members, as well as the disease burdens to the society. It would also be ideal for media campaigns and educational programs in these countries consider a LGBT individual’s own health, the health of his/her partners, and sexual freedom all together to reduce the risk for HIV spreading.

Lastly, findings of this study indicate a big role of the press in the effort to control the transmission of HIV and to end the AIDS epidemic by 2030. Results from social marketing and HIV/AIDS prevention research indicate that although the effect of media message on behavior of each individual may not be large, mass media is well known for its broad reach and great capability in re-shaping core social values and norms, personal attitudes and beliefs, leading to behavior change [52–55]. Findings of this study have demonstrated that at the country level, the practice of press freedom can interact with LGBT freedom to substantially weaken the positive relationship between LGBT freedom and HIV infection. As suggested earlier in this paper, more research is needed to examine potential underlying mechanisms by which press freedom interacts with LGBT freedom to reduce the risk for HIV infection in different countries with different cultural and societal backgrounds.

There are limitations to this study. First, findings of this study were based on an ecological model and country-level data, thus no causal conclusion can be warranted at the individual persons’ level. More in-depth analyses are needed to delineate the role of press freedom and LGBT freedom in HIV infection at the individual level – a key research topic for global health studies in the future [56, 57]. Second, the country-level press freedom is not an accurate measure of individual persons’ beliefs in the freedom of expression. Third, the measurement of LGBT freedom is semi-quantitative and is more arbitrary than objective, and further, the interval from one LGBT-FI level to another might not be equal. Fourth, other covariates at the individual level may also play a role in HIV infection, such as religious beliefs and cultural and social factors other than press freedom and LGBT freedom. These limitations further underscore the need for multi-country studies with data collected from individual participants for more comprehensive and more accurate measures of all the key variables.

Another limitation is that findings of this study cannot explain the phenomenon that a country can have high PLWH prevalence even though LGBT expression and relationships are illegal, such as several North African countries. HIV/AIDS is a multi-faceted epidemic [58–60], and free expression represents only one factor. We cannot ignore the impact of many other factors, such as treatment as prevention [61], as well as many evidence-based educational and behavioral intervention programs [62]. Data error could be another issue. The high PLWH prevalence in high PFI countries could be due to the fact that these countries have more resources to detect HIV infection; although adjustment was made for data collection in the reported the resource-limited low- and middle-income countries.

Despite the limitations, this study is the first to document the relationship between press freedom/LGBT freedom and the prevalence of PLWH with data from 148 countries, representing 96.7% of the world’s population. Findings of this study extend our understanding of the HIV epidemic to include core cultural values, demonstrating the urgency for and providing data supporting studies with individual-level data, and underscoring the need to expand the current strategies against the AIDS epidemic.

## Additional file

Additional file 1: Appendix Table. List of countries with population, persons living with HIV (PLWH), prevalence rate of PLWH (/1000), press freedom index (PFI), LGBT freedom (LGBT-F), and per capita GDP (US dollar) (DOCX 30 kb)

## Abbreviations

AIDS: Acquired Immunodeficiency Syndrome
CDC: Center for Disease Control and Prevention
CI: Confidence Interval
GDP: Gross Domestic Product
HIV: Human Immunodeficiency Virus
LGBT-FI: LGBT Freedom index
MSM: Men Who Have Sex With Men
PFI: Press Freedom Index
PLWH: Persons Living with HIV
UNAIDS: Joint United Nations Programme on HIV/AIDS
WHO: World Health Organization
