# Supplementary material for: Cross-country Association of Press Freedom and LGBT freedom with prevalence of persons living with HIV: implication for global strategy against HIV/AIDS
Source: Glob Health Res Policy. 2018 Feb 9;3:6. doi: 10.1186/s41256-018-0061-3 (PMC5806491; doi:10.1186/s41256-018-0061-3)
Supplement: Supplementary file 1 — Appendix Table. List of countries with population, persons living with HIV (PLWH), prevalence rate of PLWH (/1000), press freedom index (PFI), LGBT freedom (LGBT-F), and per capita GDP (US dollar) (DOCX 30 kb) [file 41256_2018_61_MOESM1_ESM.docx]

Appendix Table. List of countries with population, persons living with HIV (PLWH), prevalence rate of PLWH (/1000), press freedom index (PFI), LGBT freedom (LGBT-F), and per capita GDP (US dollar)

| Country | Population (1000) | PLWH  (1000) | Prevalence rate | PFI | LGBT-F | Per capita GDP (US$) |
| --- | --- | --- | --- | --- | --- | --- |
| Afghanistan | 32527 | 6.9 | 0.212 | 37.44 | 1.5 | 594 |
| Algeria | 39667 | 8.8 | 0.222 | 36.63 | 3 | 4,154 |
| Angola | 25022 | 320.0 | 12.789 | 37.84 | 3 | 4,101 |
| Argentina | 43417 | 110.0 | 2.534 | 26.11 | 5 | 13,467 |
| Armenia | 3018 | 3.6 | 1.193 | 28.43 | 4 | 3,489 |
| Australia | 23781 | 27.0 | 1.135 | 17.03 | 4 | 56,290 |
| Austria | 8611 | 18.0 | 2.090 | 10.85 | 4 | 43,636 |
| Azerbaijan | 9651 | 11.0 | 1.140 | 58.41 | 4 | 5,497 |
| Bahamas | 388 | 8.1 | 20.875 | n/a | 4 | 22,817 |
| Bangladesh | 160996 | 9.6 | 0.060 | 42.95 | 2 | 1,211 |
| Barbados | 284 | 2.6 | 9.148 | n/a | 3 | 15,429 |
| Belarus | 9513 | 35.0 | 3.679 | 47.98 | 4 | 5,754 |
| Belgium | 11286 | 20.0 | 1.772 | 11.98 | 5 | 40,454 |
| Belize | 359 | 3.6 | 10.020 | 18.54 | 4 | 4,879 |
| Benin | 10880 | 69.0 | 6.342 | 29.24 | 4 | 762 |
| Bhutan | 775 | 1.0 | 1.291 | 32.65 | 3 | 2,656 |
| Bolivia | 10725 | 18.0 | 1.678 | 31.29 | 4 | 3,077 |
| Botswana | 2262 | 350.0 | 154.697 | 22.91 | 3 | 6,360 |
| Brazil | 207848 | 830.0 | 3.993 | 31.93 | 5 | 8,678 |
| Bulgaria | 7178 | 3.9 | 0.543 | 32.91 | 4 | 6,993 |
| Burkina Faso | 18106 | 95.0 | 5.247 | 23.79 | 4 | 590 |
| Burundi | 11179 | 77.0 | 6.888 | 42.93 | 3 | 277 |
| Cambodia | 15578 | 74.0 | 4.750 | 40.99 | 4 | 1,159 |
| Cameroon | 23344 | 620.0 | 26.559 | 39.63 | 2 | 1,217 |
| Canada | 35852 | 75.5 | 2.106 | 10.99 | 5 | 43,316 |
| Cape Verde | 521 | 3.2 | 6.148 | 20.69 | 4 | 3,080 |
| Central African Republic | 4900 | 120.0 | 24.488 | 33.84 | 4 | 323 |
| Chad | 14037 | 170.0 | 12.110 | 40.17 | 4 | 776 |
| Chile | 17948 | 32.0 | 1.783 | 23.00 | 4 | 13,416 |
| China | 1371220 | 850.0 | 0.620 | 73.55 | 4 | 8,069 |
| Colombia | 48229 | 150.0 | 3.110 | 39.08 | 5 | 6,056.1 |
| Congo, DR | 77267 | 370.0 | 4.789 | 44.31 | 4 | 456.1 |
| Costa Rica | 4808 | 10.0 | 2.080 | 12.26 | 4 | 11,260.1 |
| Croatia | 4224 | 1.2 | 0.284 | 26.12 | 4 | 11,592.9 |
| Cuba | 11390 | 22.0 | 1.932 | 70.21 | 4 | 7,650.2 |
| Czech Republic | 10551 | 2.1 | 0.199 | 11.62 | 4 | 17,556.9 |
| Côte d'Ivoire | 22702 | 460.0 | 20.263 | 30.45 | 4 | 1,399.0 |
| Denmark | 5676 | 6.1 | 1.075 | 8.24 | 5 | 53,014.6 |
| Djibouti | 888 | 9.4 | 10.587 | 71.04 | 4 | 1,945.1 |
| Dominican | 10528 | 68.0 | 6.459 | 27.31 | 4 | 6,468.5 |
| Ecuador | 16144 | 29.0 | 1.796 | 33.65 | 4 | 6,205.1 |
| Egypt | 91508 | 11.0 | 0.120 | 50.17 | 2 | 3,614.7 |
| El Salvador | 6127 | 20.0 | 3.264 | 23.66 | 4 | 4,219.4 |
| Equatorial Guinea | 845 | 27.0 | 31.950 | 66.23 | 4 | 14,439.6 |
| Eritrea | 5169 | 14.0 | 2.708 | 84.86 | 3 | n/a |
| Estonia | 1312 | 13.5 | 10.290 | 11.19 | 4 | 17,084.5 |
| Ethiopia | 99391 | 793.7 | 7.986 | 41.83 | 3 | 619.2 |
| Fiji | 892 | 1.0 | 1.121 | 31.28 | 4 | 4,960.5 |
| Finland | 5482 | 2.9 | 0.529 | 7.52 | 5 | 42,403.5 |
| France | 66808 | 160.0 | 2.395 | 21.15 | 5 | 36,352.5 |
| Gabon | 1725 | 47.0 | 27.242 | 31.38 | 4 | 8,266.4 |
| Gambia | 1991 | 21.0 | 10.548 | 44.50 | 2 | 471.5 |
| Georgia | 3679 | 9.6 | 2.609 | 27.70 | 4 | 3,757.1 |
| Germany | 81413 | 73.0 | 0.897 | 11.47 | 4 | 41,178.5 |
| Ghana | 27410 | 270.0 | 9.850 | 15.50 | 3 | 1,369.7 |
| Greece | 10824 | 16.0 | 1.478 | 31.01 | 4 | 18,007.0 |
| Guatemala | 16343 | 55.0 | 3.365 | 37.92 | 4 | 3,903.5 |
| Guinea | 12609 | 120.0 | 9.517 | 28.70 | 2 | 531.3 |
| Guinea-Bissau | 1844 | 41.0 | 22.230 | 28.70 | 4 | 573.0 |
| Guyana | 767 | 7.8 | 10.168 | 27.21 | 3 | 4,127.4 |
| Haiti | 10711 | 130.0 | 12.137 | 25.08 | 4 | 818.3 |
| Honduras | 8075 | 20.0 | 2.477 | 39.27 | 4 | 2,528.9 |
| Hungary | 9845 | 4.1 | 0.416 | 27.44 | 4 | 12,365.6 |
| Iceland | 331 | 1.0 | 3.023 | 13.87 | 5 | 50,722.0 |
| India | 1311051 | 2,100.0 | 1.602 | 40.49 | 2 | 1,593.3 |
| Indonesia | 257564 | 690.0 | 2.679 | 40.75 | 3.5 | 3,346.5 |
| Iran | 79109 | 73.0 | 0.923 | 72.32 | 1 | n/a |
| Ireland | 4641 | 7.8 | 1.681 | 11.20 | 5 | 61,093.7 |
| Israel | 8380 | 8.5 | 1.014 | 32.09 | 3 | 35,729.4 |
| Italy | 60802 | 140.0 | 2.303 | 27.94 | 4 | 29,993.1 |
| Jamaica | 2726 | 29.0 | 10.639 | 11.18 | 3 | 5,105.8 |
| Kazakhstan | 17544 | 23.0 | 1.311 | 52.46 | 4 | 10,510.0 |
| Kenya | 46050 | 1,500.0 | 32.573 | 32.07 | 2 | 1,376.7 |
| Kyrgyzstan | 5957 | 8.1 | 1.360 | 30.69 | 4 | 1,103.2 |
| Laos | 6802 | 11.0 | 1.617 | 71.25 | 4 | 1,818.4 |
| Latvia | 1978 | 6.8 | 3.437 | 18.12 | 4 | 13,654.8 |
| Lebanon | 5851 | 2.4 | 0.410 | 31.81 | 2 | 8,047.6 |
| Lesotho | 2135 | 310.0 | 145.198 | 28.36 | 4 | 1,067.0 |
| Liberia | 4503 | 30.0 | 6.662 | 30.78 | 3 | 455.9 |
| Lithuania | 2910 | 1.5 | 0.515 | 18.80 | 3 | 14,251.8 |
| Luxembourg | 570 | 1.0 | 1.755 | 13.61 | 5 | 99,717.7 |
| Madagascar | 24235 | 48.0 | 1.981 | 27.43 | 4 | 401.8 |
| Malawi | 17215 | 980.0 | 56.926 | 26.41 | 2 | 372.0 |
| Malaysia | 30331 | 92.0 | 3.033 | 43.29 | 2 | 9,768.3 |
| Mali | 17600 | 120.0 | 6.818 | 36.33 | 4 | 724.3 |
| Malta | 431 | 0.5 | 1.159 | 24.16 | 4 | 22,567.9 |
| Mauritania | 4068 | 14.0 | 3.442 | 25.27 | 1 | n/a |
| Mauritius | 1263 | 8.2 | 6.494 | 27.69 | 3 | 9,252.1 |
| Mexico | 127017 | 200.0 | 1.575 | 43.69 | 4.5 | 9,005.0 |
| Mongolia | 2959 | 0.5 | 0.169 | 25.25 | 4 | 3,967.8 |
| Montenegro | 622 | 0.5 | 0.744 | 34.63 | 4 | 6,408.4 |
| Morocco | 34378 | 24.0 | 0.698 | 39.19 | 2 | 2,878.2 |
| Mozambique | 27978 | 1,500.0 | 53.614 | 29.98 | 4 | 529.2 |
| Myanmar | 53897 | 220.0 | 4.082 | n/a | 2 | 1,161.5 |
| Namibia | 2459 | 210.0 | 85.406 | 12.50 | 3 | 4,673.6 |
| Nepal | 28514 | 39.0 | 1.368 | 32.71 | 4 | 743.3 |
| Netherlands | 16937 | 22.1 | 1.305 | 9.22 | 5 | 44,290.9 |
| New Zealand | 4596 | 2.9 | 0.631 | 10.06 | 5 | 37,808.0 |
| Nicaragua | 6082 | 9.9 | 1.628 | 27.94 | 4 | 2,086.9 |
| Niger | 19899 | 49.0 | 2.462 | 23.85 | 4 | 359.0 |
| Nigeria | 182202 | 3,500.0 | 19.209 | 34.09 | 1.5 | 2,671.7 |
| Norway | 5196 | 4.5 | 0.866 | 7.75 | 5 | 74,481.8 |
| Pakistan | 188925 | 100.0 | 0.529 | 50.46 | 2 | 1,434.7 |
| Panama | 3929 | 17.0 | 4.327 | 28.98 | 4 | 13,268.1 |
| Papua New Guinea | 7619 | 40.0 | 5.250 | 25.87 | 2 | n/a |
| Paraguay | 6639 | 17.0 | 2.561 | 33.74 | 4 | 4,081.0 |
| Peru | 31377 | 66.0 | 2.103 | 31.21 | 4 | 6,027.1 |
| Philippines | 100699 | 42.0 | 0.417 | 41.19 | 4 | 2,904.2 |
| Poland | 37999 | 35.0 | 0.921 | 12.71 | 4 | 12,558.9 |
| Portugal | 10349 | 48.0 | 4.638 | 17.11 | 5 | 19,222.9 |
| Moldova | 3554 | 18.0 | 5.065 | 27.85 | 4 | 1,848.1 |
| Romania | 19832 | 16.0 | 0.807 | 24.90 | 4 | 8,980.7 |
| Russian | 144097 | 73.0 | 0.507 | 44.97 | 3 | 9,329.3 |
| Rwanda | 11610 | 200.0 | 17.227 | 56.57 | 4 | 697.3 |
| Senegal | 15129 | 46.0 | 3.040 | 27.77 | 2 | 899.6 |
| Serbia | 7098 | 3.5 | 0.493 | 27.66 | 4 | 5,237.3 |
| Sierra Leone | 6453 | 51.0 | 7.903 | 28.47 | 3 | 653.1 |
| Singapore | 5535 | 4.9 | 0.894 | 45.87 | 3 | 52,888.7 |
| Slovakia | 5424 | 0.5 | 0.092 | 11.66 | 4 | 16,089.0 |
| Slovenia | 2064 | 1.0 | 0.485 | 20.55 | 4 | 20,728.9 |
| Somalia | 10787 | 30.0 | 2.781 | 72.31 | 1.8 | 549.3 |
| South Africa | 54957 | 7,000.0 | 127.372 | 22.06 | 5 | 5,718.2 |
| South Sudan | 12340 | 180.0 | 14.587 | 38.04 | 3 | 730.6 |
| Spain | 46418 | 150.0 | 3.231 | 19.95 | 5 | 25,684.7 |
| Sri Lanka | 20966 | 4.2 | 0.200 | 60.28 | 3 | 3,926.2 |
| Sudan | 40235 | 56.0 | 1.392 | 72.34 | 3 | 2,414.7 |
| Suriname | 543 | 3.8 | 6.998 | 18.20 | 4 | 9,485.3 |
| Swaziland | 1287 | 220.0 | 170.944 | 47.28 | 2 | 3,200.1 |
| Sweden | 9799 | 9.1 | 0.929 | 9.47 | 5 | 50,585.3 |
| Switzerland | 8287 | 20.0 | 2.413 | 13.85 | 4 | 80,999.3 |
| Tajikistan | 8482 | 16.0 | 1.886 | 36.19 | 4 | 925.9 |
| Thailand | 67959 | 440.0 | 6.474 | 40.07 | 4 | 5,814.8 |
| Togo | 7305 | 110.0 | 15.059 | 28.50 | 3 | 559.6 |
| Trinidad and Tobago | 1360 | 11.0 | 8.088 | 22.39 | 3 | 17,321.9 |
| Tunisia | 11108 | 2.6 | 0.234 | 38.68 | 2 | 3,822.4 |
| Turkey | 78666 | 5.5 | 0.070 | 44.16 | 4 | 9,125.7 |
| Uganda | 39032 | 1,500.0 | 38.430 | 31.65 | 2 | 705.3 |
| Ukraine | 45198 | 220.0 | 4.867 | 39.10 | 4 | 2,115.0 |
| UK | 65138 | 101.2 | 1.554 | 20 | 5 | 43,929.7 |
| Tanzania | 53470 | 1,400.0 | 26.183 | 28.09 | 2 | 879.0 |
| USA | 321419 | 1,242.0 | 3.864 | 24.41 | 5 | 56,115.7 |
| Uruguay | 3432 | 10.0 | 2.914 | 15.94 | 5 | 15,573.9 |
| Uzbekistan | 31300 | 33.0 | 1.054 | 61.14 | 2 | 2,132.1 |
| Venezuela | 31108 | 110.0 | 3.536 | 40.61 | 4 | n/a |
| Vietnam | 91704 | 260.0 | 2.835 | 72.63 | 4 | 2,110.9 |
| Yemen | 26832 | 9.2 | 0.343 | 66.36 | 1 | 1,406.3 |
| Zambia | 16212 | 1,200.0 | 74.020 | 34.35 | 2 | 1,304.9 |
| Zimbabwe | 15603 | 1,400.0 | 89.728 | 39.19 | 2 | 924.1 |

**Note**: (1) LGBT-F: LGBT freedom: 1 = death penalty for same-sex intercourse, 2 = imprisonment, including life in prison for same-sex intercourse, 3 = expression and practice of same-sex relationships not recognized, restricted or illegal, 4 = same-sex relationships including civil union and cohabitation are recognized or legally allowed; 5= same-sex marriage is either recognized or legal. (2): n/a: not available.
